# Nanoscale spatial mapping of mechanical properties through dynamic atomic force microscopy

**DOI:** 10.3762/bjnano.10.132

**Published:** 2019-07-03

**Authors:** Zahra Abooalizadeh, Leszek Josef Sudak, Philip Egberts

**Affiliations:** 1 Department of Mechanical and Manufacturing Engineering, University of Calgary, 40 Research Place NW, Calgary, Alberta T2L 1Y6, Canada

**Keywords:** atomic force microscopy, contact resonance (CR) AFM, elastic modulus mapping, force modulation microscopy (FMM), highly oriented pyrolytic graphite (HOPG), mechanical properties, surface science, surface steps

## Abstract

Dynamic atomic force microscopy (AFM) was employed to spatially map the elastic modulus of highly oriented pyrolytic graphite (HOPG), specifically by using force modulation microscopy (FMM) and contact resonance (CR) AFM. In both of these techniques, a variation in the amplitude signal was observed when scanning over an uncovered step edge of HOPG. In comparison, no variation in the amplitude signal was observed when scanning over a covered step on the same surface. These observations qualitatively indicate that there is a variation in the elastic modulus over uncovered steps and no variation over covered ones. The quantitative results of the elastic modulus required the use of FMM, while the CR mode better highlighted areas of reduced elastic modulus (although it was difficult to convert the data into a quantifiable modulus). In the FMM measurements, single atomic steps of graphene with uncovered step edges showed a decrease in the elastic modulus of approximately 0.5%, which is compared with no change in the elastic modulus for covered steps. The analysis of the experimental data taken under varying normal loads and with several different tips showed that the elastic modulus determination was unaffected by these parameters.

## Introduction

In recent years, the study of the size-dependent properties of materials, and in particular those at the nanometer scale, have received significant attention [1–3]. More specifically, through interrogation of materials at this small length scale, the development of advanced materials that have significantly improved mechanical [1], electrical [4], and bio-compatibility [5] properties have been enabled. Focusing on mechanical properties of materials, the nanoscale mechanical properties, including elastic and shear moduli, can drastically differ from their bulk values. This results in opportunities to custom design or enhance bulk engineering materials with advances discovered through nanoscale interrogation [1]. Thus, quantitative measurements of nanoscale mechanical properties can offer important insights into material functionalities at the nanoscale with high temporal resolution [6–7]. Furthermore, mechanical properties measured at the nanometer scale are critical for the development and verification of predictive models of failure, specifically when the failure mode occurs at a nanoscale interface of two different phases, such as those in composite materials [8–9]. Therefore, the study of nanoscale mechanical properties is critical for the development of next-generation materials, advanced mechanical systems, and predictive models of failure.

Dynamic atomic force microscopy (AFM) is one technique that is well suited for experimentally measuring the mechanical properties of materials with high spatial resolution [10–12]. More specifically, a focus on two dynamic AFM modes, force modulation microscopy (FMM) and contact resonance (CR) AFM, will allow for the overarching goal of nanoscale mechanical property measurements to be realized. In FMM, the tip is pressed into contact with the surface and oscillated at a frequency off resonance. The obtained variations in the measured amplitude of the cantilever are associated to the elastic modulus through a straight forward calculation [13]. In CR mode, the mechanical properties can be extracted by pressing the tip into the surface and oscillating the cantilever at a frequency corresponding to the contact resonance, which corresponds to the mechanical resonance of the cantilever and tip in contact with the sample surface. In this mode, the variation in the amplitude and shifts in the contact resonance frequency during scanning are recorded. While the obtained shift in the contact resonance frequency can be converted into a quantitative map of mechanical properties, the surface displacement map in CR mode can qualitatively reflect the elastic modulus with higher sensitivity as compared to other dynamic modes [14].

The FMM and CR AFM techniques are widely used for mapping the local, nanoscale elastic properties of polymers, rubber, composites, and biological samples. Moreover, in comparison to static mode AFM or other experimental techniques used to measure the mechanical properties of materials, such as nanoindentation, the material property maps resulting from FMM and CR modes can have high spatial resolution and can be acquired at relatively high speeds. Of course the advantages of dynamic AFM come at the expense that the lateral shear force between the tip and sample cannot be eliminated, making the technique inappropriate for weakly bonded samples. On the other hand, more variations of the experimental set up are possible for AFM, such as ultrahigh vacuum (UHV) AFM, which can increase the spatial resolution of the measured mechanical properties as well as the reliability of the measurements made.

In this work, both FMM and CR modes have been employed to examine surfaces of highly oriented pyrolytic graphite (HOPG) under UHV conditions in order to spatially resolve mechanical property variations resulting from atomic-scale surface defects on the HOPG crystal. In this manuscript, we will elaborate the advantages of these specific settings and the importance of the outcome. The surfaces are examined under UHV conditions to exclude environmental contaminants on the surface when measuring the mechanical properties of atomic-sized defects [15–17]. Furthermore, the high quality factor of the AFM cantilever that is achieved under UHV conditions can be very beneficial in dynamic AFM modes, as the Q-factor is inversely proportional to the force sensitivity [18]. Thus the sensitivity to the measurement of mechanical property variations on the surface can be increased when measured under UHV conditions. Both FMM and CR modes have been employed in this study: while quantitative determination of the elastic modulus using FMM is more reliable, greater contrast in the variation of the amplitude of the oscillating cantilever can be captured in the CR mode over atomic-scale defects that exhibit mechanical properties variations [14,19].

Given the intense examination of HOPG as an ideal lubricant [20] and a surface that has been extremely well-characterized in both experiments and simulations [21–23], the examination of this surface with dynamic AFM will allow for verification that both FFM and CR modes can be used to capture atomic-scale variations in mechanical properties. Furthermore, examining the mechanical properties of the HOPG surface can verify predictions of how the surface behaves under sliding conditions [14]. A previous study of tip convolution on HOPG surfaces [24] illustrated a possible change in the elastic modulus over HOPG steps, but they could not clarify their idea because of the lack of experimental data. Additionally, there have been several studies, particularly in the field of tribology, that have attributed observations or proposed mechanisms of friction that result from a weaker elastic constant at an atomic step edge [21,24–25]. Despite the number of proposed mechanisms relying on weakened graphite step edges, there has never been an experimental measurement of their mechanical properties. Thus, one objective of this paper is to experimentally determine if there is a variation in the mechanical properties of HOPG atomic steps, providing conclusive experimental evidence to the previous theoretical assumptions made about HOPG and graphene step edges to interpret friction and AFM tip-convolution measurements made previously.

In this paper, CR AFM is used to clearly identify atomic-scale defects, such as atomic step edges, that show mechanical property variations on surfaces of HOPG. FMM is then used to scan the surface and provide quantifiable measurements on the variation of the elastic modulus. The results are then compared with other techniques to quantify the elastic modulus of HOPG. Additionally, the influence of the normal force and tip size are examined as well as the influence of the oscillation frequency of two FMM and CR modes on the response amplitude.

## Experimental

All experiments were conducted in an RHK 7500 UHV-AFM system operated under ultrahigh vacuum conditions (pressure less than 5 × 10^−10^ Torr) and at room temperature. Atomically flat samples of HOPG were prepared by mechanically cleaving the sample in laboratory air, then immediately placing them in the fast entry lock of the UHV system, and pumping the fast entry lock down within 10 min of cleaving the samples. Once the fast entry lock reached a pressure of 1 × 10^−7^ Torr, the fast entry lock chamber and transfer arm were baked at 120 °C for two hours. Subsequently, the HOPG samples were transferred into the AFM chamber, where two dynamic mode AFM experiments were conducted.

Silicon probes with an integrated tip (Nanosensors PPP-CONT) were used as force sensors. The normal bending and the lateral twisting spring constants of the force sensor were determined under ultrahigh vacuum using the beam-geometry method, involving the measurement of the frequency of the first normal oscillation mode to determine the thickness of the cantilever [26]. The normal stiffness of the cantilevers was determined to be in the range of 0.25–0.45 N/m and the lateral stiffness was between 80–140 N/m. The optical sensitivity of the quadrant detector was assumed to be the same in both the lateral and normal direction and was determined by measuring the slope of the cantilever normal bending signal versus sample displacement in the *z*-direction. The zero applied normal force was determined by measuring the average deflection of the cantilever when the tip was far from the HOPG surface. The lateral force is defined as the measured twisting signal of the cantilever during scanning.

**Figure 1:**
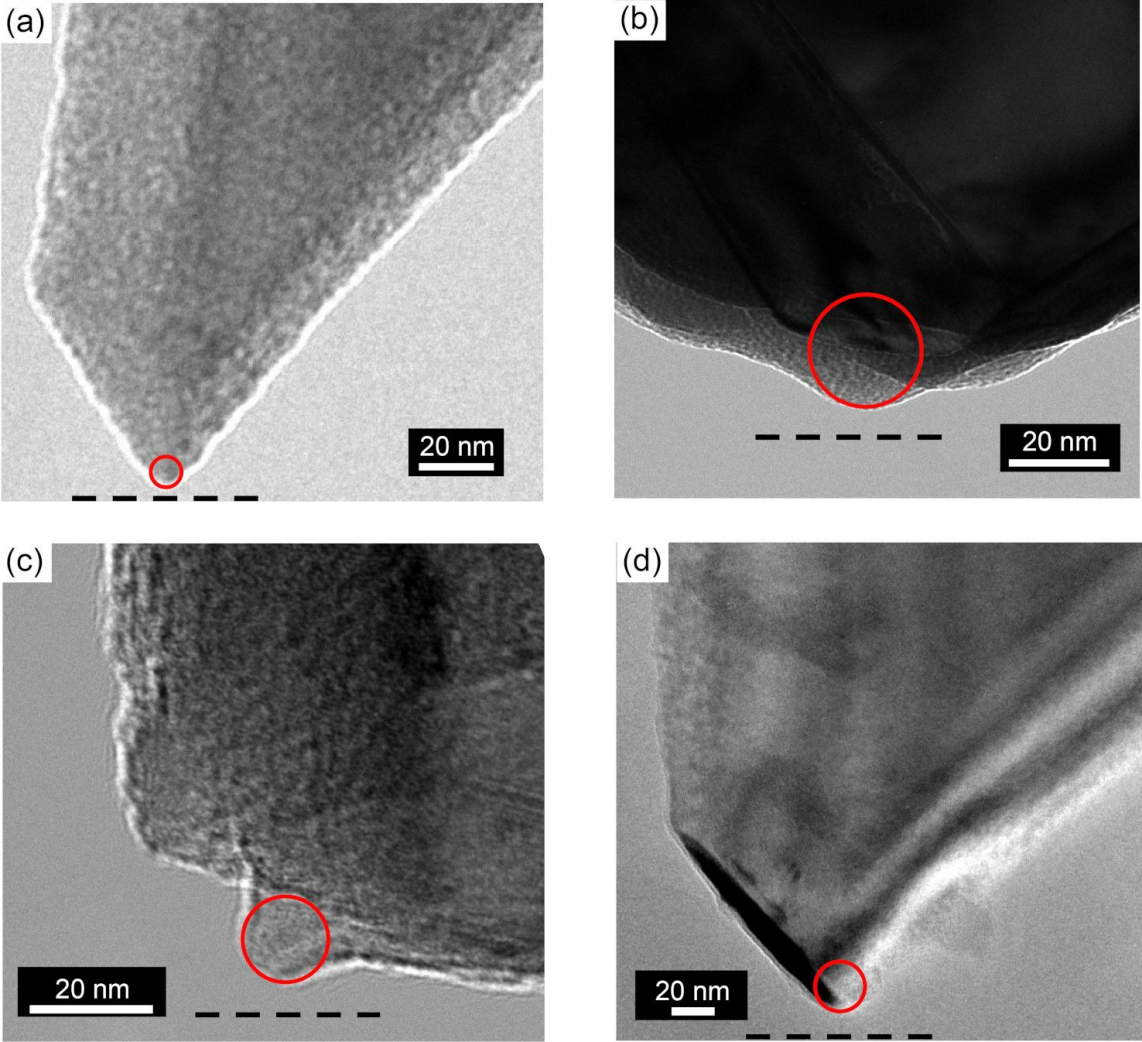
TEM image of four different tips employed in the dAFM experiments. The tips are referred to as (a) tip A, (b) tip B, (c) tip C, and (d) tip D in their acquired images. A circle in a red solid line has been drawn to emphasize the selected radius of each tip. Each TEM image has been rotated to compensate for the tilt angle of the AFM cantilever with respect to the plane of the sample surface, thus making a horizontal line in this figure the plane of the surface. The measured tip radii are as follows: (a) 3.0 ± 0.2 nm, (b) 25 ± 5 nm, (c) 10 ± 1 nm, and (d) 15 ± 1 nm and the adhesion force corresponding to the tips is as follows: (a) 4.0 nN, (b) 8 nN, (c) 5.5 nN, and (d) 6 nN. The black dashed line in the bottom indicates the HOPG surface plane.

Post-mortem images of the tip apex were captured with a Tecnai F20 transmission electron microscope (TEM) using a custom designed sample holder based on the design reported in [27]. Profiles of the lowest asperity were measured, accounting for the 22° tilt of the cantilever with respect to the sample surface in the RHK beetle-type beam deflection AFM. The tip images were taken after scanning the HOPG surface so that the tip size at the last moment of scanning could be determined. Figure 1a–d shows TEM images of four different tips utilized in collecting the experimental data. The tips will be referred to as tip A (Figure 1a), tip B (Figure 1b), tip C (Figure 1c), and tip D (Figure 1d) in the corresponding acquired AFM images within this manuscript. In all cases, the tip radius was observed to vary between 5–25 nm following all experiments.

### Contact resistance (CR) AFM experiment

**Figure 2:**
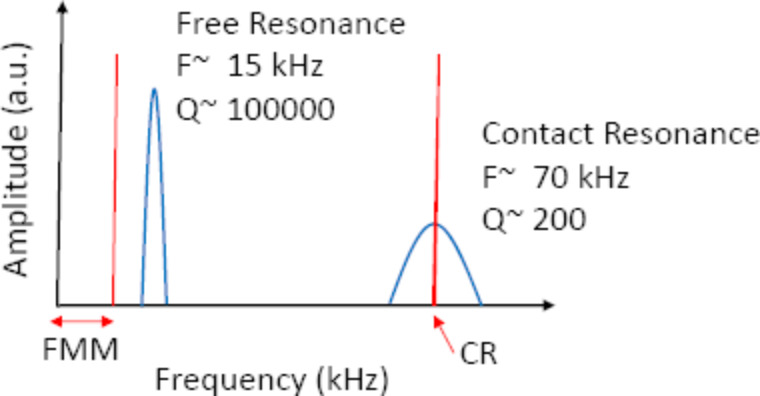
Schematic of the non-contact/free resonance and contact resonance frequencies of the AFM cantilever used in all experiments (blue lines). Typical frequencies and Q-factors that are measured both in non-contact (*f* = 15 kHz, *Q* = 100,000) and in contact mode (*f* = 70 kHz, *Q* = 200) are shown on the graph. Excitation frequencies used in CF-AFM and FMM modes are shown with red lines. The FMM measurement has a range of excitation frequency which is smaller than the first free resonance of the cantilever and above 1 kHz.

The first set of experiments on the HOPG surface were conducted in qualitative imaging CR mode AFM to obtain the highest sensitivity. In this mode the cantilever was placed in contact with the sample surface at a constant mean deflection or normal force while acquiring an image. A modulation of the cantilever deflection was superimposed by shaking the cantilever with a constant vertical amplitude and at the first contact resonance frequency that was not varied or tracked during scanning. The relative resonance frequencies of the cantilever out of contact, in contact, and the moduluation frequency used in CF AFM are shown in Figure 2.

The contact resonant frequency was determined by placing the tip in contact with the surface on an HOPG terrace at the desired force to be used when acquiring an image. The cantilever was then excited with a constant amplitude and the frequency linearly increased, such that the resonance could be identified. This procedure was repeated before scanning at a new normal force. The typical contact resonance frequencies measured ranged from 70–85 kHz. Oscillating the cantilever at its contact resonance frequency allowed for greater sensitivity in detecting the cantilever oscillation for a given excitation amplitude [28–29]. Along the modulated frequency, a constant oscillation amplitude of approximately 1 nm determined when the tip was far from the HOPG surface was used in all measurements. This 1 nm oscillation amplitude measured far from the surface typically resulted in an amplitude that was less than 5 Å when the tip was placed in contact with the HOPG surface. The HOPG surface was scanned under a constant preload force, modulation frequency, and drive signal while the amplitude and phase response of the cantilever were recorded simultaneously with the topography and the lateral force signals using a digital lock-in amplifier supplied with the RHK R9 controller.

### FMM experiment

In the second set of experiments, the AFM was operated in the FMM mode on the HOPG surface. For FMM the cantilever was placed in contact with the sample surface at a constant mean deflection while acquiring an image. A modulation of the cantilever deflection was superimposed by shaking the cantilever with a constant vertical amplitude and at very low frequencies (<10 kHz). Moving towards a lower frequency than the CF-AFM mode allows for determination of the quantitative mechanical properties and ensures that the resonance peaks do not interfere with the amplitude response. The off-resonance measurement was conducted at 1.5 kHz over the surface. This change in excitation frequency between CR and FMM modes is highlighted in Figure 2. While the FMM measurements were conducted over a wide spectrum range (as long as the narrow frequency bands of the contact resonance are avoided) in previous studies, the elastic modulus calculation by FMM requires operation in regions of the spectrum where there is little or no change in amplitude for small frequency shifts. This is to ensure that the amplitude response is restricted to variations of the sample elasticity. Along the modulated frequency, a constant oscillation amplitude of approximately 1 nm determined when the tip was far from the HOPG surface. This was applied in all measurements. The HOPG surface was scanned under a constant preload force and a constant drive signal while the amplitude and phase response of the cantilever were simultaneously recorded with the topography and the lateral force signals using a digital lock-in amplifier supplied with the RHK R9 controller.

The calibration of the detected variations in amplitude and phase into elastic and loss modulus with high accuracy requires a good knowledge of the employed dynamic mode. We focus on converting the obtained surface displacement into elastic modulus in this manuscript. Here, the conversion of the measured oscillation voltage signal (surface displacements) to nanometers was realized by the optical sensitivity of the quadrant detector as follows:

[1] A(m)=A(v)∗S(m/v)

where *A*(*m*) is the calibrated amplitude signal in meters, *A*(*v*) is the measured amplitude signal in volts from the lock-in amplifier, and *S* is the sensitivity of the photodetector measured from the linear portion of a force versus distance curve.

The force applied to the surface can be separated into two contributions: the applied compressive and adhesive forces exerted when the tip is in contact with the surface as measured by the static bending of the cantilever, and the oscillatory perturbations of the excited cantilever. For the oscillatory perturbation, the drive force exerted on the cantilever was first determined by measuring the amplitude response on a sample that is considered to be infinitely stiff relative to the cantilever [13]. In this measurement, the frequency was set to the same oscillation frequency used during scanning and the measurement was performed on the surface of a standard silicon chip. The obtained amplitude response on a silicon surface, *A*_Silicon_ can be used to calculate the drive force exerted on the tip as:

[2] $$F_{\text{dr}}=k_{\text{n}}\cdot A_{\text{Silicon}}$$

where *F*_dr_ is the drive force, *k*_n_ is the normal stiffness of the cantilever obtained from geometric methods [26], and *A*_Silicon_ is the cantilever oscillation amplitude determined on a silicon surface. In the next step we brought the cantilever in contact with the HOPG surface without changing the cantilever excitation frequency and amplitude to maintain the same drive force. The drive force was maintained constant during the scanning of the HOPG surface.

Knowing the amplitude response over the surface allows one to determine the elastic modulus of the surface through several specific steps of modeling and calculations. The first step involves the conversion of the measured amplitude response to the effective normal stiffness [10], while effective stiffness is the reduced stiffness of the cantilever and contact. This conversion was accomplished by modeling the cantilever stiffness in series with the contact stiffness that represents the boundary conditions at the tip–sample contact. The conversion of the measured oscillation amplitude to an effective stiffness was accomplished with the following equation:

[3] $$F_{\text{dr}}=k_{\text{eff}}\cdot A_{\text{HOPG}}$$

where *F*_dr_ is the drive force obtained on the silicon surface, *A*_HOPG_ is the amplitude response of the cantilever measured along the HOPG surface, and *k*_eff_ is the effective stiffness.

In the next step, the contact stiffness, required for the determination of the elastic modulus, must be deconvolved from the effective stiffness using the following equation:

[4] $$\frac{1}{k_{\text{eff}}}=\frac{1}{k_{\text{n}}}+\frac{1}{k_{\text{con}}}$$

where *k*_n_ is the normal stiffness of the cantilever and *k*_con_ is the normal contact stiffness.

The next step is to determine the effective elastic modulus by employing the calculated contact stiffness. A logical approach is to apply the basic equations of a contact mechanics model to determine the mechanical properties. In principle either the Hertzian [30] or Derjaguin–Muller–Toporov (DMT) models [31] can be employed to calculate the mechanical properties of the contact. However, this approach requires precise knowledge of micro- and nanoscale quantities that are difficult to measure accurately. A more practical technique that has been shown to yield accurate results is a relative or comparative approach [32–33]. In this approach, the mechanical properties are determined through a normalization against a material with known mechanical properties. The mechanical properties of the calibration sample are considered to be known, either through independent measurements with another technique or from literature values. To obtain the effective elastic modulus, we adopted the mechanical properties from literature. By employing the normalization equation as below, the mechanical properties of the sample were determined.

[5] $$E_{\text{test}}^{*}=E_{\text{cal}}^{*}{\left(\frac{\alpha_{\text{test}}}{\alpha_{\text{cal}}}\right)}^{\frac{3}{2}}$$

In this equation, the exponent *m* = 3/2 denotes a Hertzian contact, α is the normalized contact stiffness 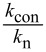 of either the test surface or calibration sample, 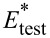 is the effective elastic modulus of the sample of unknown modulus, 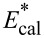 is the effective elastic modulus of the calibration sample, α_test_ is the normalized contact stiffness of the unknown sample, and α_cal_ is the normalized contact stiffness of the calibration sample. The calibration sample in these calculations was a polycrystalline Nb glass sample. The reported modulus for polycrystalline Nb is 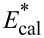 = 125 GPa and the normalized contact stiffness is α_cal_ = 80 [14].

Finally, once the effective elastic modulus of the HOPG sample was determined, the elastic modulus of the HOPG sample was generated using the following equation:

[6] $$\frac{1}{E^{*}}=\frac{1-\nu_{\text{t}}^{2}}{E_{\text{t}}}+\frac{1-\nu_{\text{s}}^{2}}{E_{\text{s}}}$$

where the *E*_t_, ν_t_, *E*_s_, and ν_s_ are the elastic modulus and Poisson’s ratio of the tip and sample, respectively. The Poisson’s ratio and the elastic modulus of the tip were 0.26 and 160 GPa [34–35]. The Poisson’s ratio of the surface was assumed to be constant in all experiments, and thus a value of 0.25 [36] was used in the determination of the elastic modulus variation of HOPG as the tip was scanned across the surface. All AFM experimental data was processed using WSXM software [37]. All results contained within this manuscript have been repeated for at least three different tips on three different areas of the HOPG surface. Finally, force modulation amplitudes in the normal direction were sufficiently small that the displacement of the cantilever in the direction parallel to the long axis of the cantilever [38] was substantially smaller than the lattice spacing of the graphite lattice. Thus, the tip remained in a single point during a single oscillation cycle used in the modulation of the normal force.

## Results

**Figure 3:**
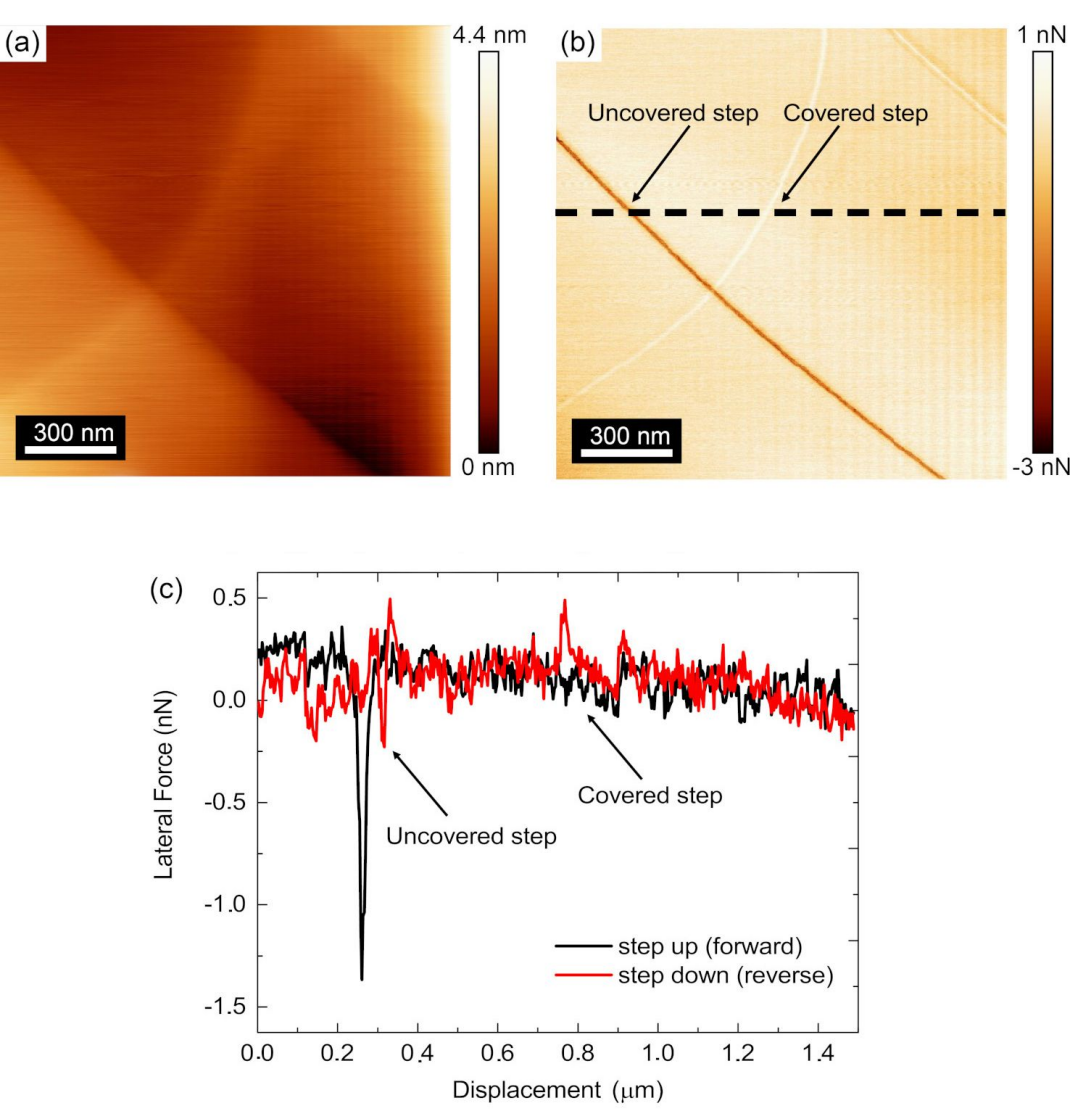
AFM (a) topographic and (b) reverse scan direction lateral force maps of the HOPG obtained by FMM. The covered and uncovered single atomic steps have been labeled in (a) to specify their locations. A dashed black line in (b) shows the location where the lateral force profile in (c) was obtained from, which crosses over both the uncovered and covered atomic steps. (c) Lateral force line profile of the forward and reverse scan directions, highlighting the difference in the forces measured for uncovered and covered steps. The data presented in this figure were acquired with tip A.

Figure 3a shows an example topographic image of a freshly cleaved graphite surface acquired with tip A. This particular image has a single atomic step which is uncovered, such that the step edge interacts directly with the sliding AFM probe, as well as a step which is covered by one or several layers of graphene, preventing a direct interaction of the step edge with the sliding AFM tip. Figure 3b shows the simultaneously recorded lateral force map. In this case, the uncovered step was observed to have darker contrast (more negative) compared with the surrounding terrace, resulting from the tip traversing the step in the step-up direction. A much fainter contrast is observed on the covered step, which shows a brighter contrast (more positive) compared to the surrounding terrace resulting from the tip traversing the terrace in the step-down direction. The measured heights over the steps were determined to be 0.33 ± 0.02 nm and 0.11 ± 0.02 nm for the uncovered and covered steps, respectively.

While some difference in the sharpness of the two steps may be observable in Figure 3a, where the uncovered step appears sharper than the covered step, this contrast difference is not as reliable as the smaller measured lateral and friction forces on the covered steps compared with the uncovered steps, as reported in [39]. Figure 3c shows the line profile extracted along the dashed line in Figure 3b for both the lateral force acquired in the forward and reverse scan directions. By observing the difference between the forward and reverse scan lateral forces, the magnitude of the friction can be observed. From the examination of the difference between the forward and reverse lateral forces at the uncovered and covered steps, it is clear that the friction is higher than the surrounding terrace at the uncovered step than for the covered step as has been extensively observed previously [15,40]. All subsequent identifications of uncovered and covered steps in the remainder of this manuscript have been identified similarly. Additionally, Figure 3c shows that the lateral force peaks have a more negative value than the values of the lateral forces measured on the terrace in both the forward and reverse scan directions. In accordance with [15], this finding suggests that the UHV environment and baking prior to surface characterization has resulted in very little water or other contamination of the exposed steps, thus allowing for the measurement of the intrinsic properties of the step edges themselves. The results contained within subsequent sections of this manuscript also showed the same trend in the lateral forces over the step edges.

### Results of contact resonance (CR) AFM experiments

**Figure 4:**
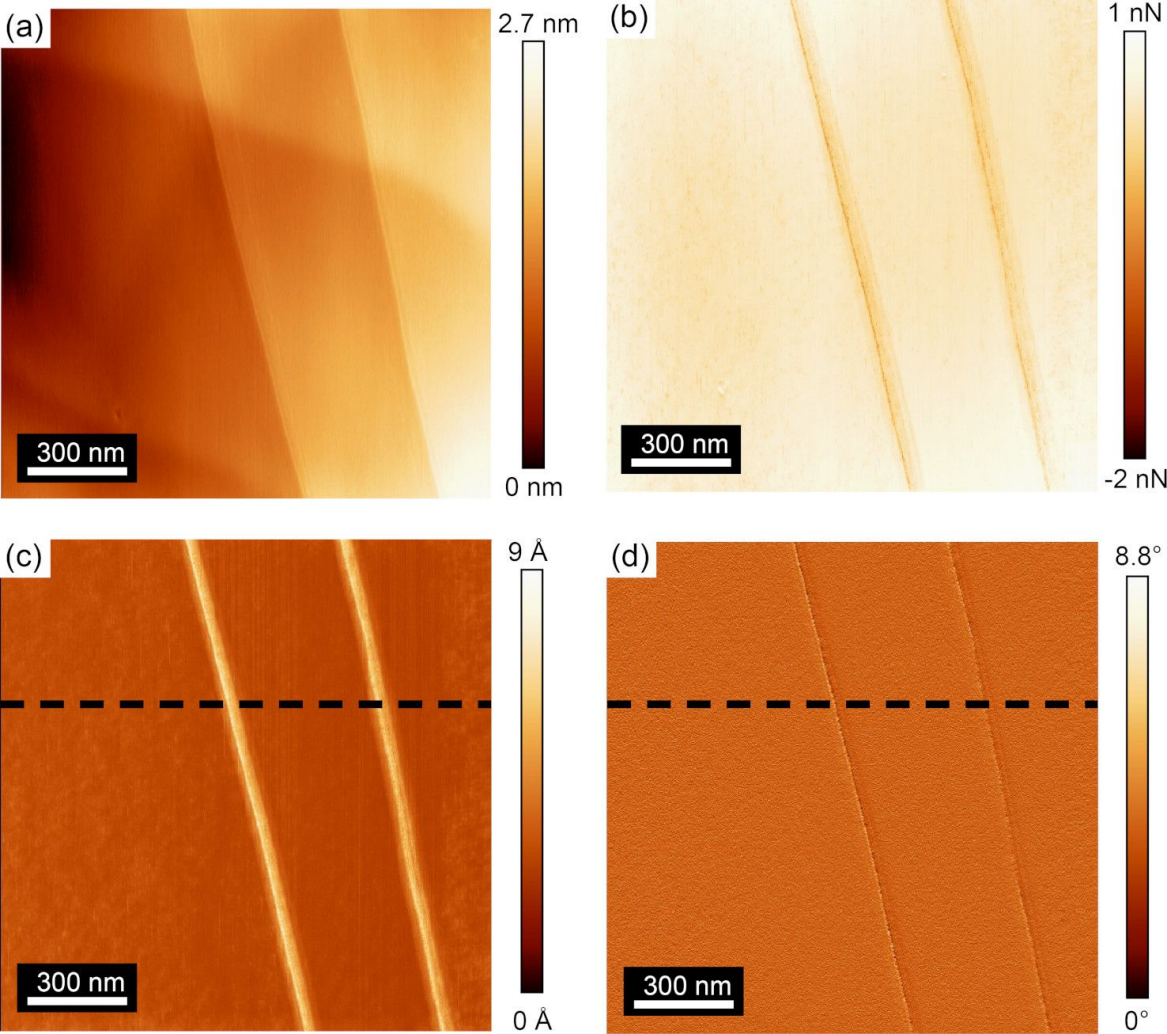
(a) Topographic and (b) lateral force maps acquired in the forward scan direction on uncovered and covered steps of an HOPG surface from the CR experiment. The simultaneously recorded (c) amplitude and (d) phase maps (modulation frequency of 82 kHz) show contrast only from the uncovered steps. The data presented in this figure were acquired with tip B.

**Figure 5:**
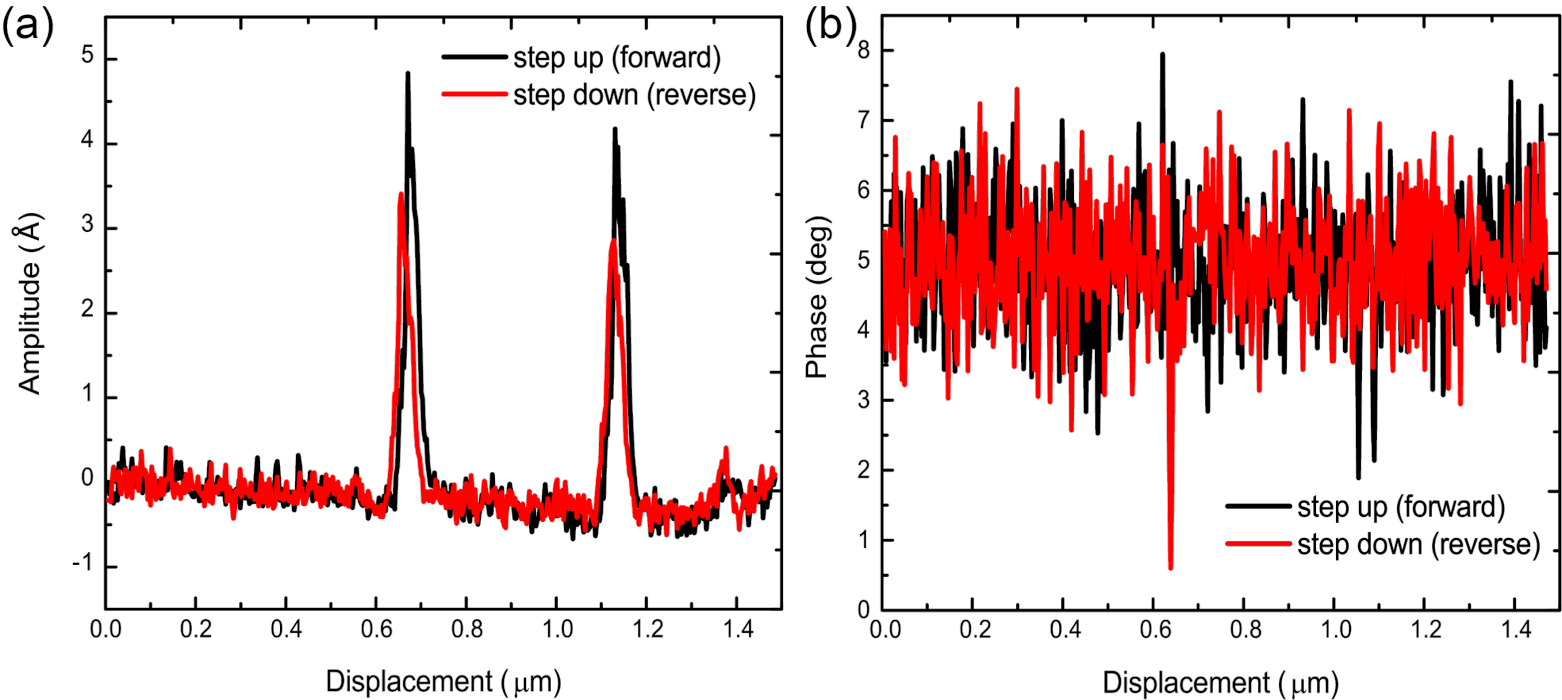
Line profile of (a) amplitude and (b) phase at uncovered steps acquired with the CR-mode at a contact resonance of 82 kHz. The data presented in this figure were acquired with tip B.

Figure 4a shows a topographic image of another region on the HOPG surface containing both uncovered and covered steps acquired with tip B. Figure 4b shows the lateral force map acquired in the reverse scan direction. The CR experiment was conducted at a contact resonance frequency of 82 kHz for higher sensitivity in amplitude and phase response. The simultaneously recorded amplitude and phase maps through the CR experiment in Figure 4c and Figure 4d, respectively, show correlating features from the steps identified in the topographic and lateral force maps. In this case, the amplitude image in Figure 4c shows a strong contrast to the uncovered steps, while there is no contrast with the covered steps. Additionally, the contrast on the uncovered steps was observed to be positive in every measurement of an uncovered step for both directions, as will be shown in Figure 5a. Figure 4d shows a very slight variation in the phase as the tip traversed the uncovered steps, but otherwise no significant change in the phase signal was observed. The lack of any significant variation in phase suggests that damping of the cantilever resulting from dissipative forces is insignificant and thus not considered in the subsequent analysis of the experimental results [14,34]. Similar variations in phase over the uncovered steps were observed for all measurements presented within this manuscript, allowing for the same assumption to be made in calibrating those results.

To gain further insight into the amplitude and phase variations that were detected over covered and uncovered steps, line profiles were extracted along the dashed black lines in Figure 4c and Figure 4d in both forward and reverse scan directions. Figure 5a shows the variation in amplitude as the tip was scanned in the forward and reverse directions. This line profile data clearly shows that the amplitude is invariant with the scan direction. Moreover, the line profile data indicates no amplitude variation over covered steps as was present in amplitude image (Figure 4c). While some contrast was observed in Figure 4d of the phase response, a closer examination of the line profile data in Figure 5b shows that no discernible peak or contrast can be detected from the uncovered or covered step edges in phase, as the noise or measured variation in the phase is the same magnitude as any variation that can be observed in the phase line profile.

The results of these CR-experiments show that there is a significant variation of the amplitude over uncovered steps while there is no amplitude variation over covered steps. This finding indicates a change in the elastic modulus on uncovered steps while no elastic modulus variation over covered steps. Alongside the amplitude variation, no change in the phase response was observed for both covered and uncovered steps. This finding indicates that there is no variation in viscosity of the HOPG across its atomic defects. Realizing these qualitative measurements, FMM was conducted to quantify the changes in the elastic modulus over uncovered steps.

### Results of force modulation microscopy (FMM) experiments

**Figure 6:**
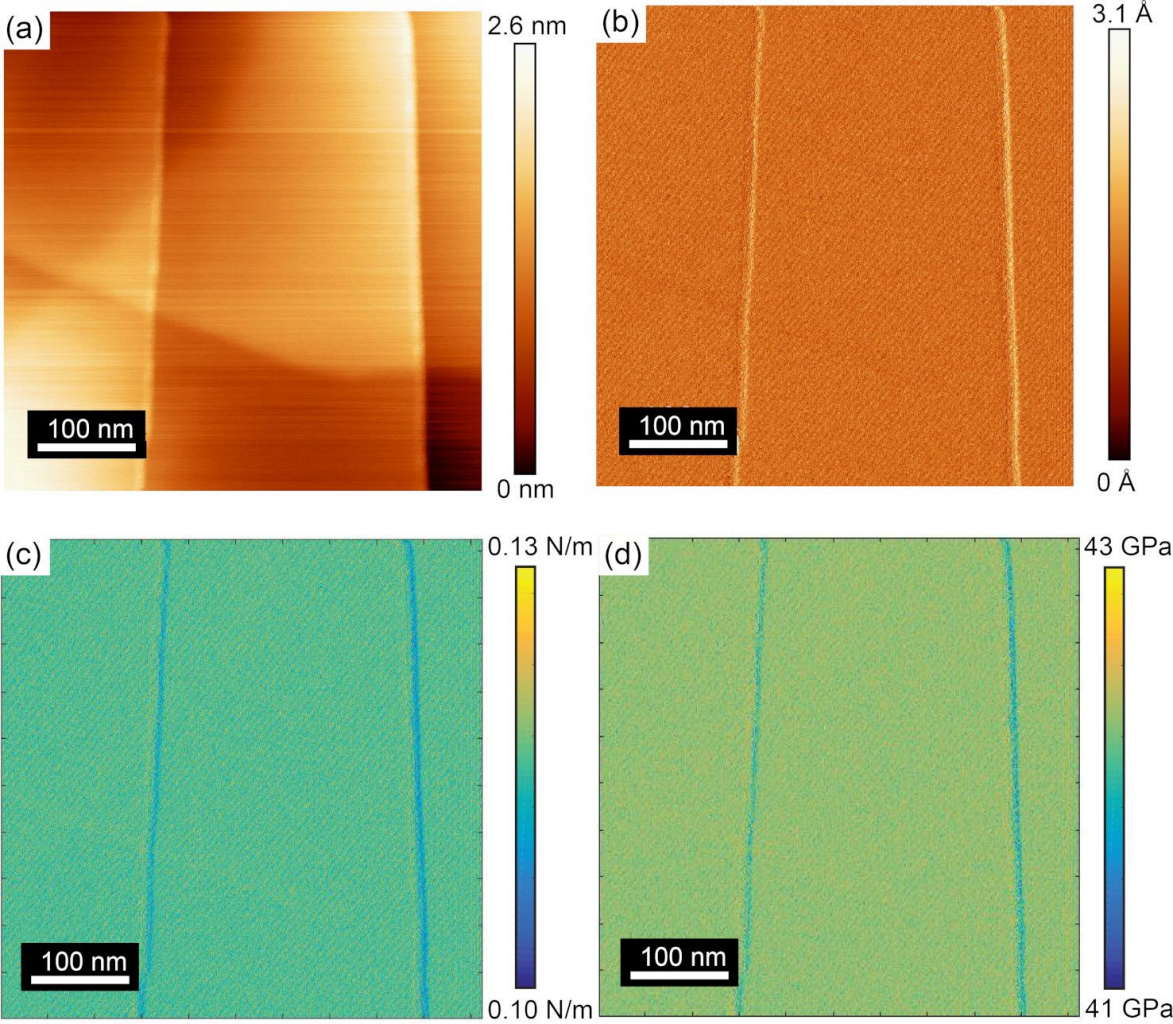
(a) AFM topographic map of a third area of the HOPG surface containing both uncovered and covered steps acquired with FMM. (b) Corresponding amplitude map (1.5 kHz modulation frequency) measured on the surface. Calculated (c) contact stiffness and (d) elastic modulus maps of the surface. The data presented in this figure were acquired with tip C.

Figure 6a shows a topographic map of a third area of the HOPG surface containing both uncovered and covered steps acquired with FMM using tip C. The FMM amplitude signal in Figure 6b shows Ångström variations in the measured amplitude signal, again showing an increase in the amplitude at the uncovered step edges and no variation over covered step edges, the same as the trend observed in the amplitude response over uncovered steps measured with the CR-experiment. The conversion of the amplitude response into contact stiffness is shown in Figure 6c and subsequently into elastic modulus in Figure 6d. As the amplitude was observed to increase for all uncovered step edges, the inverse relation between amplitude and contact stiffness/elastic modulus necessitates that the step edges must be softer than the surrounding terrace.

The measured elastic modulus over the terrace in Figure 6d was observed to be 41.50 ± 0.08 GPa, where the error reported represents the standard deviation in the measured elastic modulus over the terrace. At the step edges, the modulus was observed to decrease by a factor of approximately 0.4%, to 41.3 GPa. Again, as there was no variation in the amplitude response measured in FMM over covered steps, the elastic modulus did not change over these defects.

## Discussion

In this section, we will compare the measured mechanical properties of HOPG with those previously published in the literature, specifically looking at how atomic defects can influence mechanical stiffness. Additionally, as the amplitude response of the cantilever measured while scanning over an uncovered atomic step of HOPG can be influenced by a number of factors, each factor will be examined individually to rule out their impact on the quantitative measurement of the elastic modulus on both the atomic terrace and the step edge. The factors that are specifically addressed in this section include: the impact of surface topography, environmental contamination, the Schwoebel–Ehrlich barrier, energy dissipation through viscoelastic energy losses, applied normal force, tip shape, and modulation frequency (both for CR and FMM modes). Each examination will show that the elastic modulus measured in the previous section is unchanged by these factors. The discussion is divided into different subsections of mechanical properties, applied normal force, tip shape, and modulated frequency while all other factors mentioned above are discussed in the first section of mechanical properties.

### Experimentally measured mechanical properties

While the mechanical properties of atomic-scale features of the HOPG surfaces have not been widely reported, several studies investigate the contact stiffness as well as the elastic modulus variation over the HOPG surface. Thus, we use the information shown in Figure 6c and Figure 6d to examine our findings on the mechanical properties of HOPG terraces with the existing literature. We first investigate our finding on contact stiffness before delving into a discussion of the elastic modulus.

The average measured contact stiffness over the HOPG flat terrace is 0.11 ± 0.05 N/m, which is comparable to previous studies where the contact stiffness on an atomic terrace was found to be 0.12 N/m [25]. A reduction of contact stiffness over the uncovered step edges is evident in Figure 6c. The contact stiffness depends on the surface topography or local roughness, which can in turn change the contact area of the tip with the surface, as well as the variation in local mechanical properties [28]. First, we address issues with local topographic variations of the surface that influence the measured contact stiffness. A reduction of the contact stiffness over the uncovered step edges can be due to a change in the contact area over the inclined steps [39]. However, examination of the covered step edges shows no variation in the response amplitude despite similar topographic variations as the uncovered steps. Therefore, we can conclude that the local variations in the surface topography do not significantly influence the contact stiffness as the tip traverses the atomic step. Summarizing the discussion, we believe that a local variation in the mechanical properties is more likely to be contributed to the measured variation in contact stiffness over the uncovered step edges than the local topographical variation.

Beyond the contact stiffness, the elastic modulus of HOPG has been investigated in both experiments and simulations [41]. The reported values range between 17 GPa [42] to 60 GPa [43]. The drastic change in the reported values depend on the employed methodology and scale of the measurements. An effective analytical molecular mechanics model of a graphene sheets [44] was employed in [41] that calculates Young’s modulus of single-walled carbon nanotubes, which extends the model for graphite platelets under infinitesimal deformation. In another study, an elastic modulus of 39.5 GPa was reported based on the force–stretching relation of carbon atoms, while another theoretical study [42] suggested an out-of-plane elastic modulus of 17 GPa for HOPG; the later study considered the thermal expansions of graphite. Here the acquired elastic modulus and its variation over the step edges of graphite falls within the broad range of reported elastic modulus of graphite.

In the FMM study of graphite, the measurements of HOPG surfaces indicate an elastic modulus of 41.5 ± 0.08 GPa over flat traces of HOPG, with a reduction of 0.2 GPa over uncovered steps, while covered steps do not exhibit any change in elastic modulus. Static indentation measurements using stiff cantilevers (≈40 N/m normal bending spring constant) were conducted and are summarized in the Appendix. These results showed that the mean elastic modulus of the HOPG terrace was 38 GPa, but could not be acquired with sufficient spatial resolution to measure the variation of the elastic modulus over an atomic step edge, as was possible with CR and FMM. However, the static indentation measurements show that the FMM calibration is consistent with well-established nanoindentation measurements conducted on the same microscope. We can also explore the literature to elucidate the reduction of elastic modulus over the uncovered step edges of graphite beyond what has been shown for the elastic modulus.

Previous research on graphene step edges shows evidence of elastic straining of graphene edges [25], which behave as nanoscale springs. This study indicated a reduction in the lateral force as the tip was dragged into the edge of a graphene step in the forward/step-up scan direction, while in the reverse/step-down direction of scanning, no change in the lateral force was observed. The increase in the lateral force was proposed to be a result of the deformation and buckling of the step edge as the tip traversed to the higher level terrace. While the observations in [25] were acquired under humid, ambient conditions, which can significantly influence the lateral forces measured at step edges [15], our measurement, under UHV conditions, shows that the softening of the step is present in both scan directions. Additionally, the trend observed in the lateral forces, where both the forward and reverse scan directions showed a more negative value in the peak lateral force at the uncovered step edge, suggests an absence of contamination at the step edge. Thus, the measured properties are a result of contaminant-free step edges.

In another study [24], the width of the uncovered step edge of the HOPG surface was evaluated in both experiment and simulation. In this study, both results were compared with the topography at the step predicted by simple rigid-body geometry. Comparison between the simulation results and the rigid body model suggested that increased elastic deformation of the step as the tip scanned over the step edge resulted in the increased step width observed in the simulations compared to that predicted by the rigid body model. The study showed that the topography of the uncovered atomic steps can be correlated to tip size and that this correlation is affected by the deformation of the atomic steps. The origin of the difference between the simulation and experimental observations in this case was a result of the atomic step bending significantly as the tip reached the end of the terrace, indicating that the mechanical stiffness of the step edge was reduced by the presence of a step edge. Our finding of a reduced elastic modulus at the step edges provides the quantifiable, experimentally observed mechanism to understand the results contained within [24]: the elastic modulus of a step edge is lower than the surrounding terrace. The finding is also consistent with the enhanced deformation mechanism at step edges used to interpret enhanced friction at graphene step edges in [25] and gives the first quantifiable measurement of this variation. We believe that this is a purely elastic phenomena, where no energy is lost through dissipative interaction of the oscillating tip during CF-AFM or FMM-AFM measurements, other than through the small amount of additional energy lost through frictional dissipation at the uncovered step edge resulting from the Schwoebel barrier [45–46] in the absence of contaminants with a rounded AFM tip as detected in the lateral forces acquired simultaneously [15]. More specifically, the elastic displacement of the step edge by the modulating normal force did not show any enhanced contrast in the acquired phase images in CR-AFM or FMM-AFM results, suggesting no additional viscoelastic energy losses from modulating the applied load during scanning at the step edge. Thus, the presence of the Schwoebel barrier did not significantly impact the mechanical property measurements. We further clarify the term “dissipative interaction” in this manuscript to be any energy loss through a viscoelastic pathway, where the same definition has been used in frequency modulation (FM) AFM studies, such as in [47], to explain energy loss through vertical elastic displacement of step edges. In this case, energy loss is a result of the additional work of displacing the step in a single oscillation, compared to a measurement on the atomic terrace, which in itself does not suggest a viscoelastic energy loss pathway.

Before moving on to the discussion of the other factors that can influence the measurement, we should point out that we have considered the viscosity of the sample and we refer the reader to the Appendix section and where the contact is modeled with viscous and spring elements. While the same measurement has been done in CR mode to calculate the elastic modulus from the the cantilever dynamics without any normalization, it can be seen from Figure 10 that the assumption of the viscous element resulted in erroneous determination of the elastic modulus on a graphite terrace.

### Influence of applied normal force

As discussed earlier, the measured amplitude response using FMM can be influenced by a number of factors, including the applied normal force. The total normal force exerted by the tip on the surface can be thought of as a combination of the applied normal force and the contact geometry, as this will influence the adhesive contribution to the total normal force. More specifically, given that the material systems are constant, the shape of the tip apex will then determine the adhesive interaction between the tip and sample. This section will examine the influence of the applied normal force on the measurement of the elastic modulus of the steps in comparison with the terraces of HOPG.

**Figure 7:**
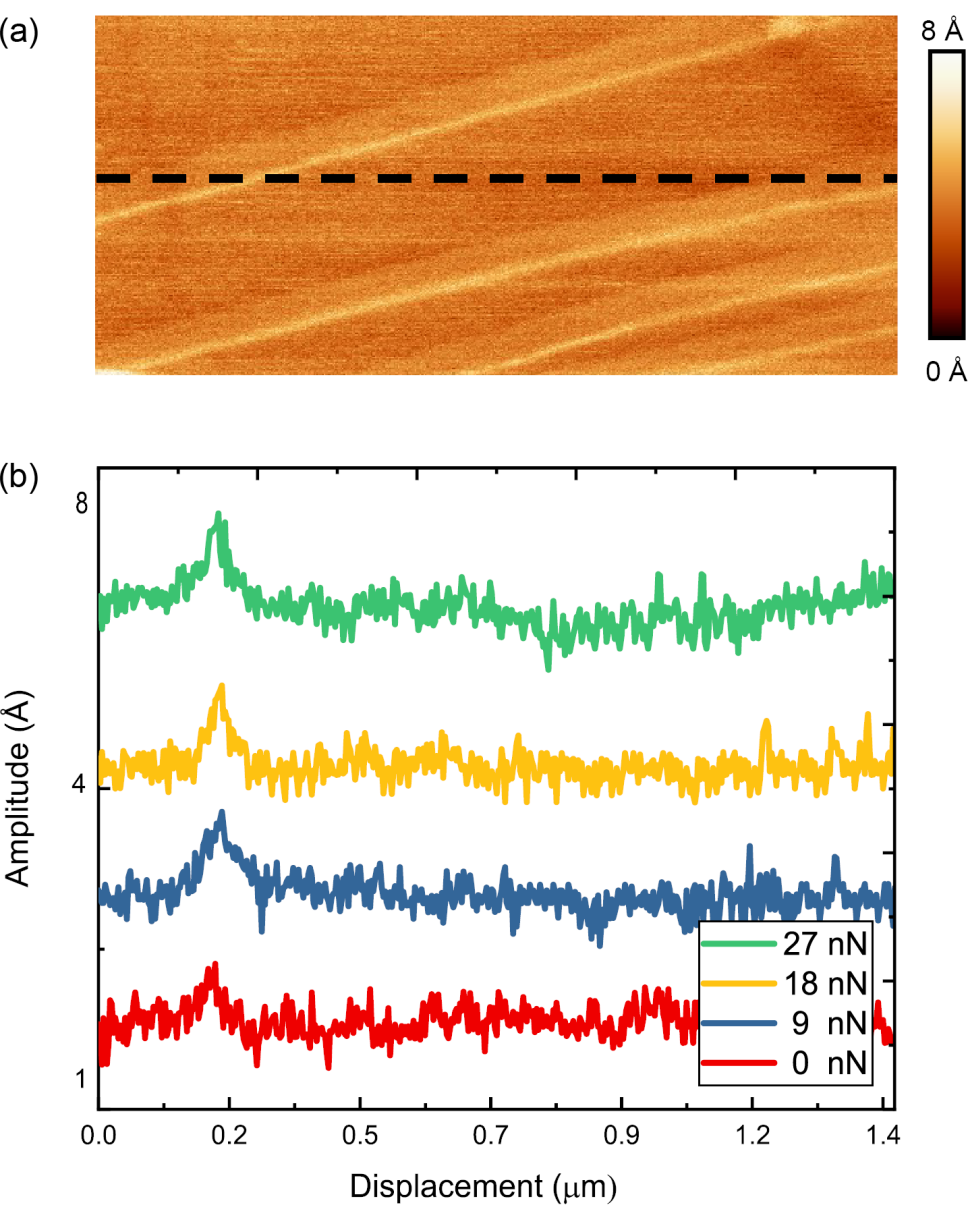
(a) Amplitude map obtained while the tip traversed four single-height, uncovered atomic steps on an HOPG surface in CR (modulation frequency = 78 kHz). (b) Amplitude line profiles acquired along the black dashed line in (a). The normal force was increased from 0 nN (red), 9 nN (blue), 18 nN (yellow), and 27 nN (green) while recording the amplitude variation. Each line profile has been offset by a value of 4 Ångström to make identification of the amplitude variation over the uncovered step clear. The data presented in this figure were acquired with tip D.

Figure 7a shows an amplitude map of the cantilever response while it traversed four single and uncovered atomic steps (image obtained with tip B). This CR measurement was conducted at the corresponding contact resonance frequency of 78 kHz. This area was scanned multiple times at a number of different loads to determine the influence of the applied compressive load on the measured amplitude response in the on resonance measurement with the higher sensitivity. As the contact resonance varies slightly with the load [48] (approximately 3 kHz within the 30 nN change in normal force in this measurement), the oscillation frequency of the drive signal was altered to maintain the contact resonance frequency for the given normal force in the same area of scanning. Figure 7b shows the amplitude line profiles acquired along the black dashed line of Figure 7a. For the range of applied loads examined in this manuscript, the amplitude response peak over the uncovered steps did not exhibit any change in the height or width of the peak. Thus, we can conclude that the variation of the applied normal force, which has significant influence on the contact area [49] and the resonant frequency [48], does not significantly influence the amplitude response as long as the measurement is conducted at the resonance frequency corresponding to the applied load. Thus, the measured softening at the atomic step edges is not impacted by the applied load.

**Figure 8:**
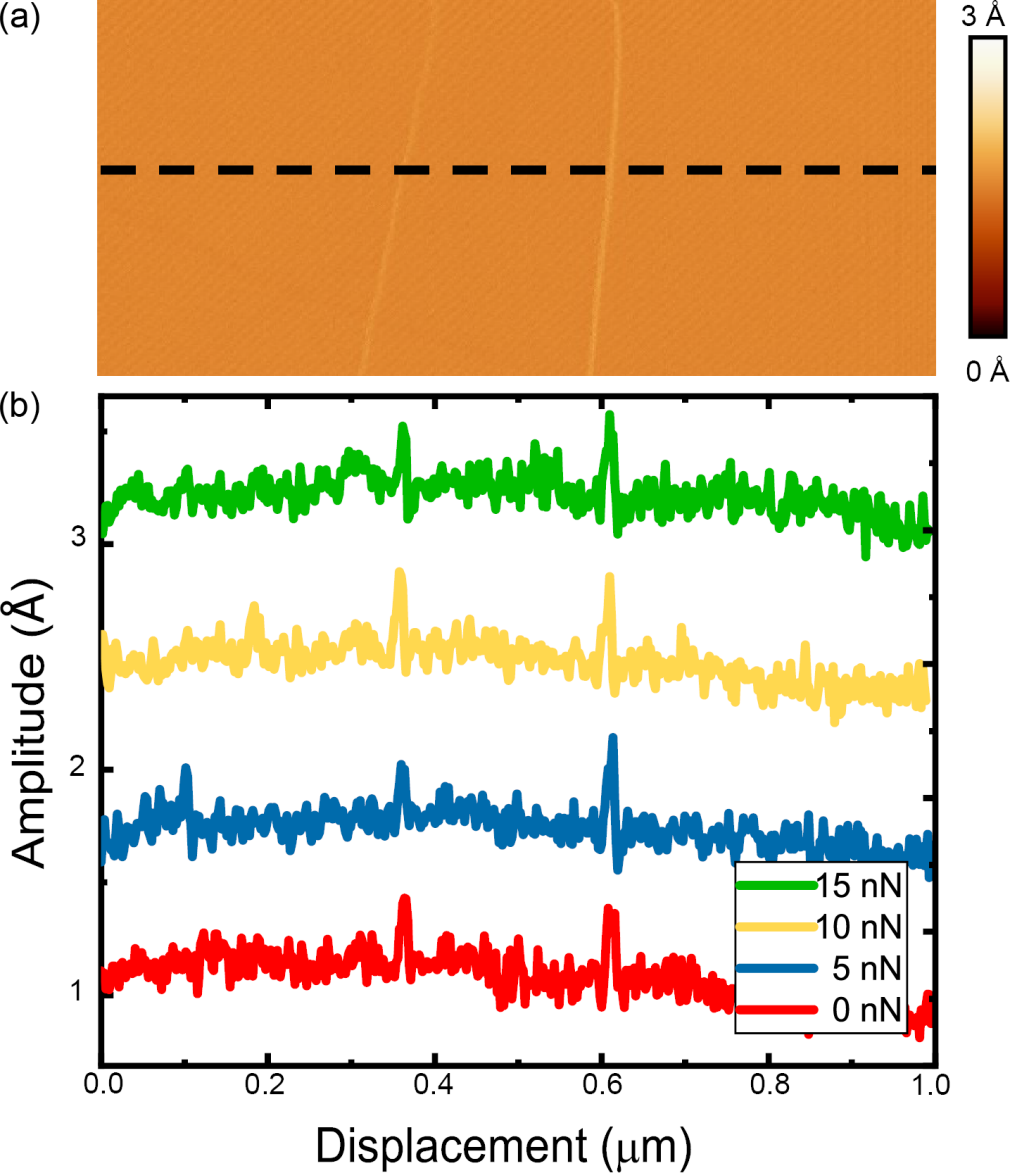
(a) Amplitude map obtained while the tip traversed two single-height uncovered atomic steps on an HOPG surface in FMM (modulation frequency = 1.5 kHz). (b) Amplitude line profiles acquired along the black dashed line in (a). The normal force was increased from 0 nN (red), 5 nN (blue), 10 nN (yellow), and 15 nN (green) while recording the amplitude variation of the cantilever. Each line profile has been offset by a value of 1 Ångström to make identification of the amplitude variation over the uncovered step clear. The data presented in this figure were acquired with tip C.

Figure 8a shows an amplitude response on the HOPG surface with two uncovered step edges (image obtained with tip C) acquired in the FMM. This measurement was conducted with a modulation frequency of 1.5 kHz, far below the cantilever contact resonance frequency. This area was scanned multiple times at a number of different loads to determine the influence of the applied compressive load on the measured amplitude response in the off-resonance measurement at the constant modulation frequency. Figure 8b shows the amplitude line profiles acquired along the black dashed line of Figure 8a. The finding in terms of the amplitude is very similar to the previously discussed result in the on-resonance case, considering the fact that this time in the off-resonance measurements all experiments at different loads have been conducted at the same frequency of 1.5 kHz. For the range of examined applied loads, the amplitude response peak over the uncovered steps did not exhibit any change in the height or width of the peak, which indicates that the amplitude response in the off-resonance measurement is constant under the variation of the applied load in the indicated range.

Thus for both FMM and CR modes, no change in the amplitude response was observed with varying normal force over the atomic steps of the HOPG surfaces. While the contact resonance frequency does depend on the applied load, and had to be adjusted during the load-dependent measurement, in-off resonance measurements, the modulation frequency was held constant at a value of 1.5 kHz, independent of the applied load. The only difference between the findings in both measurements is that the on-resonance frequency measurements have higher sensitivity and contrast in the amplitude maps compared to the off-resonance measurements.

As a final note, as was mentioned in the previous paragraph, the contact resonance measurements showed significantly higher variation in the amplitude as the tip traversed over the surface. Despite the higher sensitivity in detecting variations in the surface displacement, the covered step did not appear in any of the on-resonance measurements under the varying normal forces. As this defect was not observed in the contact resonance measurements, we believe that one layer of graphene is sufficient to mask the mechanical property variations that result from the low coordinated atoms of the step beneath. Thus, while adhesion [50] and other mechanical properties like friction [51] can be influenced by covered steps of the HOPG surface, out-of-plane elasticity is not.

### Influence of tip shape

As discussed previously, the size and shape of the tip will influence the adhesive interaction between the tip and sample, and thus the total applied normal force. As we have labeled the measurements shown within this manuscript, we can follow the effect of tip shape on the determination of the mechanical properties of the surface. Figure 7 and Figure 8 show the amplitude response that was obtained with tip D (CR-AFM) and tip C (FMM), respectively. Through comparison of the two measurements, one can see that the amplitude response shows greater contrast for the on-resonance measurement (CR-AFM) compared to the off-resonance measurement (FMM) when traversing a step edge. These two measurements indicate that the major contributing influence to the measurement of mechanical properties could be either the tip shape or the modulation frequency. To focus more on the influence of tip radius, a comparison must be drawn between the various CR measurements shown in Figure 4 and Figure 7, where measurements have been conducted with tip B and D, respectively. The tip apex radius changes from 25 nm in tip B to 15 nm for tip D. Despite this change in tip size, the response amplitude between the terrace and the step edge is the same.

We also repeated the calibration of the elastic modulus of the terrace and the step edges for two additional tips beyond what was shown for tip C in this manuscript, labeled tip E and F. Table 1 shows that the elastic modulus determination for both the terrace and the step edge is the same for all three tips, which had a variation in tip radius of over 11 nm. Thus, as stated above, the amplitude variation in CR AFM was unchanged with tip radius, as well as the elastic modulus determination for FMM. We thus conclude that tip radius does not influence the determination of the mechanical properties of the substrate, and that the repetition of measurements with the various tips have yielded the same measurement of the elastic modulus over the surface within the experimental errors reported.

**Table 1:** The elastic modulus measured from FMM measurements of graphite surfaces and uncovered atomic steps from three different tips.

Tip	Elastic modulus of terrace (GPa)	Elastic modulus of steps (GPa)	Tip radius (nm)

C	41.50 ± 0.08	41.31	10 ± 1
E	41.51 ± 0.06	41.32	14 ± 1
F	41.57 ± 0.05	41.31	21 ± 3

### Influence of modulation frequency

As mentioned before, we deliberately employed two different modulation frequencies in the study of atomic defects. We have conducted the experiment at resonance as well as off resonance for higher sensitivity and calculation purposes, respectively. The influence of the modulation frequency can be observed in the magnitude of the response amplitude in Figure 7 and Figure 8 where the measurements have been conducted on-resonance and off-resonance, respectively. The amplitude response has a higher magnitude in the on-resonance measurements compared to the off-resonance measurements, which is evident by the larger contrast observed in Figure 7a compared with Figure 8a. Given that the tip radius has been excluded as a contributing factor to the variation in amplitude measured, the last remaining factor responsible for the observed sensitivity to mechanical properties is the modulation frequency. Thus, care must be taken when choosing the modulation frequency.

## Conclusion

Dynamic AFM measurements were conducted on HOPG surfaces. Both FMM and CR AFM experimental results showed an increase in the amplitude response of the AFM cantilever as the tip slid between two atomic terraces of the surface, indicating a lower elastic modulus at the step edge. In both cases, only uncovered step edges showed an amplitude variation and covered steps showed no variation in amplitude. Thus, topographic contributions to variations in the measured amplitude could be excluded as a contributing factor to elastic modulus maps produced using dynamic AFM. CR-AFM showed a larger variation in amplitude over the step edges compared with FMM AFM. The quantification of the elastic modulus on the HOPG terrace was performed using FMM AFM and through analysis of the dynamics of the cantilever. Through the relative modulus determination on Nb glass using FMM, values for the elastic modulus on HOPG terraces were obtained that are similar to those reported in the literature. Using this relative modulus calibration technique, the modulus on the terrace was determined to be 41.5 ± 0.08 GPa. The reduced elastic modulus measured over the uncovered step edge was determine to be 41.3 GPa. The analysis of the applied normal force, tip shape, and modulation frequency showed that almost no variation was observed in the elastic modulus determination or the contrast in amplitude images when the normal force or the tip radius was changed. The FMM results also confirm the mechanisms that have been proposed for enhanced friction at step edges that result from a weakening of the modulus in this region.

## Appendix

**Figure 9:**
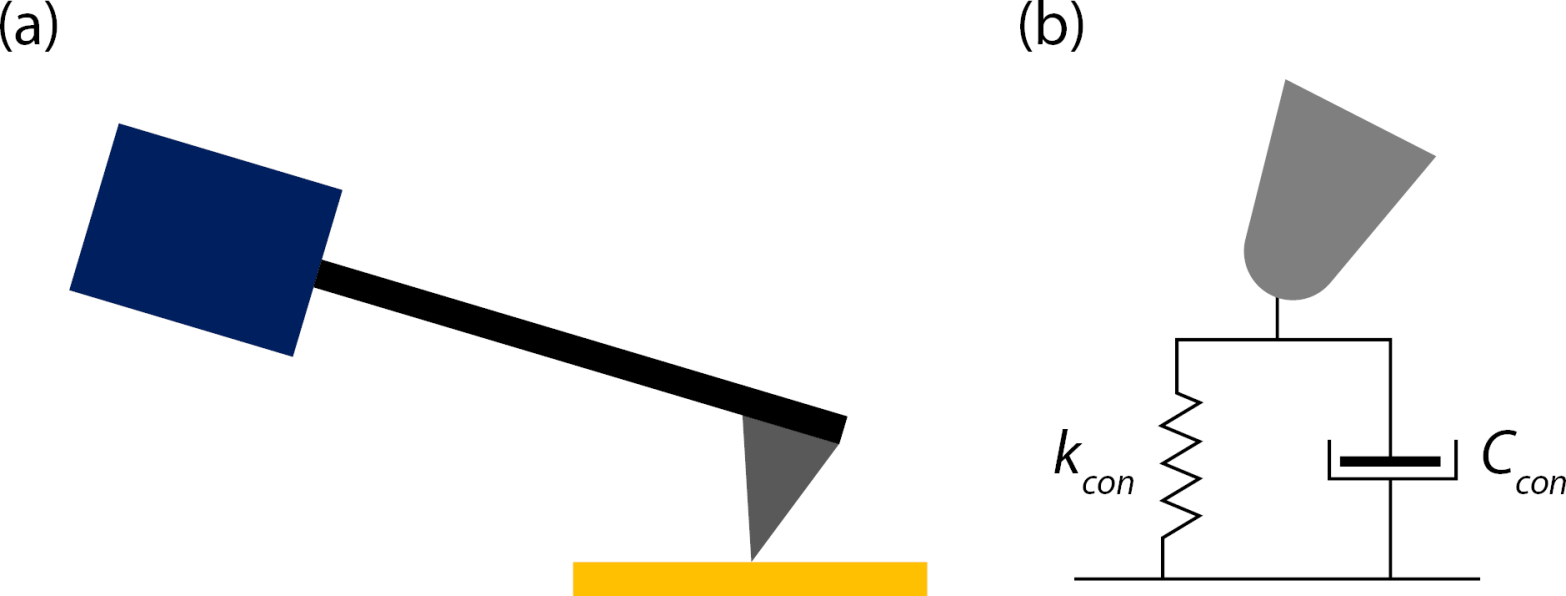
(a) Schematic of the physical construction of the cantilever. A piezo actuator (blue rectangle) excites the cantilever/tip assembly that is in contact with a sample (yellow rectangle). (b) A Kelvin–Voigt model representing the tip–sample contact in dynamic AFM mode.

We considered a nonlinear sample response by measuring both the viscoelastic and elastic aspects of the contact when examining the data collected without using a relative calibration procedure. We modeled the contact with a highly sophisticated model where the Kelvin–Voigt model can represent the boundary conditions at the tip–sample contact. Thus, the cantilever spring is in series with the contact elements, which are two Kelvin–Voigt linear elements with a spring, accounting for the contact stiffness and a dash pot describing the contact damping. Figure 9 illustrates two elements of a spring and a dash pot in parallel, defining the contact at the tip of the cantilever and the sample.

**Figure 10:**
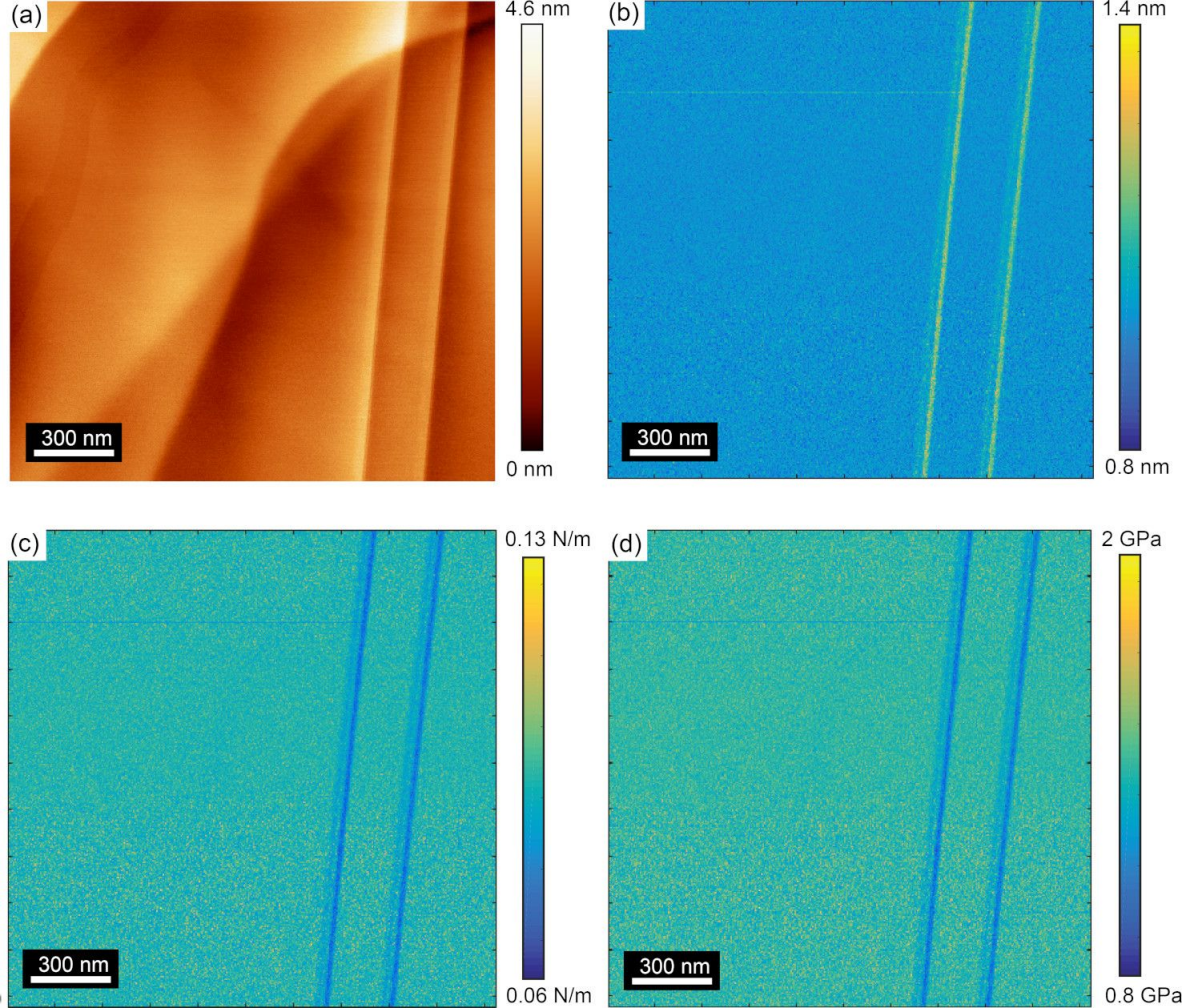
(a) AFM topography image, (b) amplitude, (c) corresponding converted contact stiffness and (d) corresponding converted elastic modulus of the HOPG surface. This series of images was recorded with tip A and with CR ARM (modulation frequency = 77 kHz.)

The elastic modulus was calculated through the equilibrium conditions of a Hertzian contact [31] and the obtained contact stiffness. However, we were unable to reproduce the published values of the elastic modulus of graphite [41–44] with the data obtained from the FMM experiments. Figure 10 indicates the calculated elastic modulus through FMM experiments and calculations described in [31]. It is possible to see the very small contribution of viscoelastic effects in Figure 5b of the manuscript, which shows almost no variation in the phase as the tip traverses up and down the uncovered and covered HOPG step edges. The calibration sample was a polycrystalline Nb glass, which also has very little viscoelasticity, similar to the HOPG sample.

As indicated in Figure 10, the calculated elastic modulus using the Kelvin–Voigt model resulted in an elastic modulus of about 2 GPa for the HOPG surface, which is far away from the reported values for the elastic modulus of HOPG. Given the finding in the Kelvin–Voigt model we believe that despite the variance in the stiffness of our calibration sample with respect to the HOPG sample, that the nonlinear sample response can be ignored and the application of relative modulus measurements contained within our manuscript are relevant and reliable. Finally, we would like to point out that the use of the relative modulus allowed for the determination of the elastic modulus of the HOPG terrace to a value very close to the reported value in the literature [41–44]. Thus, we believe that our subsequent report of a 0.5 percent decrease in the elastic modulus at atomic step edges is also very accurate.

To further understand and interpret our FMM results, we conducted static indentation measurements on the graphite surface with stiffer AFM cantilevers (PPP-NCL), having a stiffness of approximately 40 N/m. The force–distance curves have been recorded and then the cantilever bending was removed from the force curve to find the force versus the displacement curve. Finally the force–distance curve was fit with a Hertzian contact to find the elastic modulus of the indentation from the fitting using Equation 7 [49].

[7] $$F=\frac{4}{3}E^{*}R^{1/2}d^{3/2}$$

**Figure 11:**
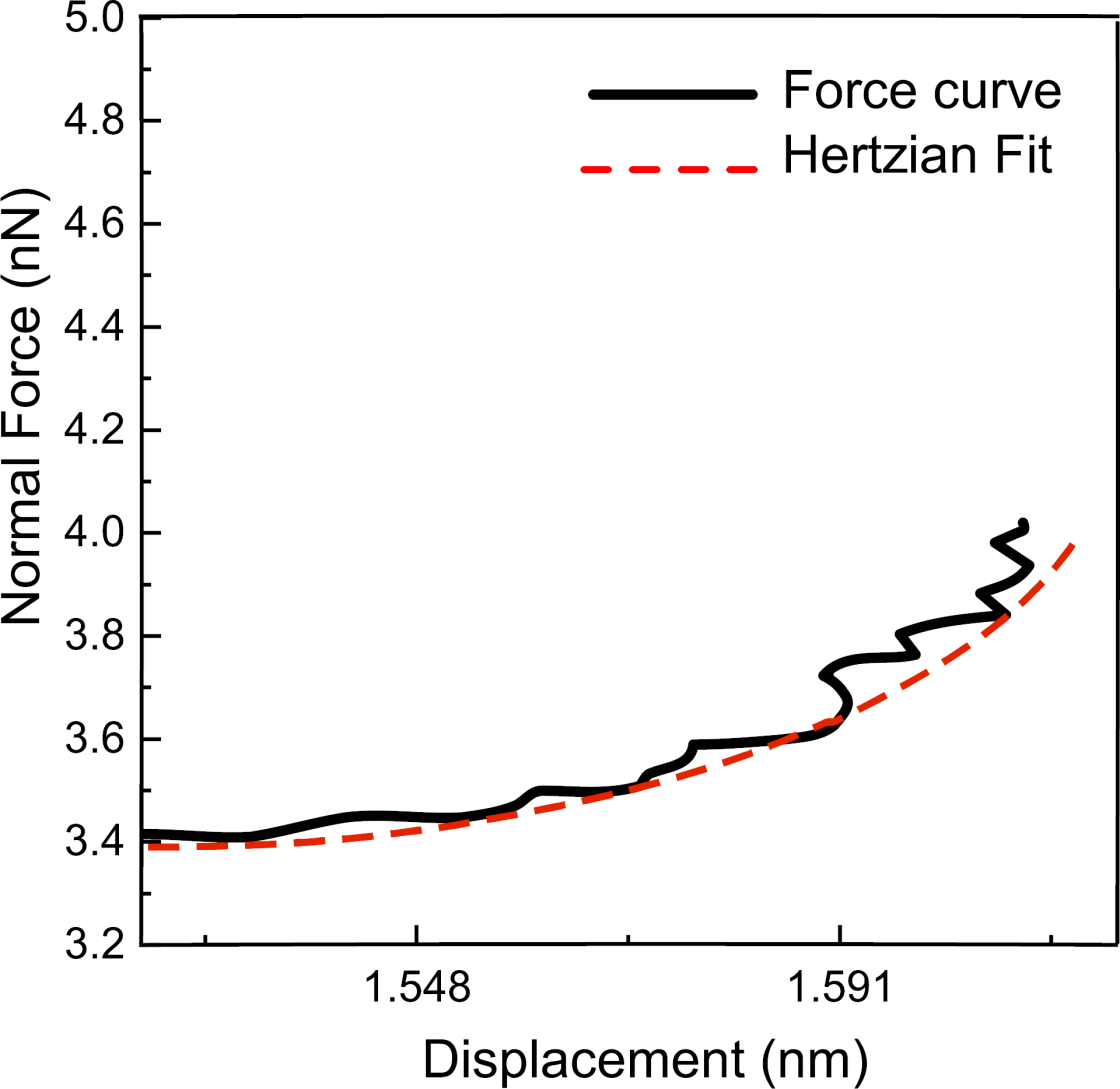
Force–distance curve from the static indentation measurement on an HOPG surface using a cantilever with a stiffness of 39 N/m. The force versus displacement (black curve) was fit with a Hertzian contact (red curve) to extract the elastic modulus. The radius of the tip apex that made contact with the surface was 11 nm.

Figure 11 indicates the force–distance curve with the Hertzian fitting, and the elastic modulus was exported from the fitting. In these measurements, we were able to extract the same value of the elastic modulus that we obtained using the softer PPP-CONT cantilevers used in the FMM imaging. Given the common elastic modulus measurement made with the two different cantilevers, it can be assumed that the low stiffness of the cantilever in the FMM measurements is not an issue for the measurement of graphite surfaces and step edges.

**Figure 12:**
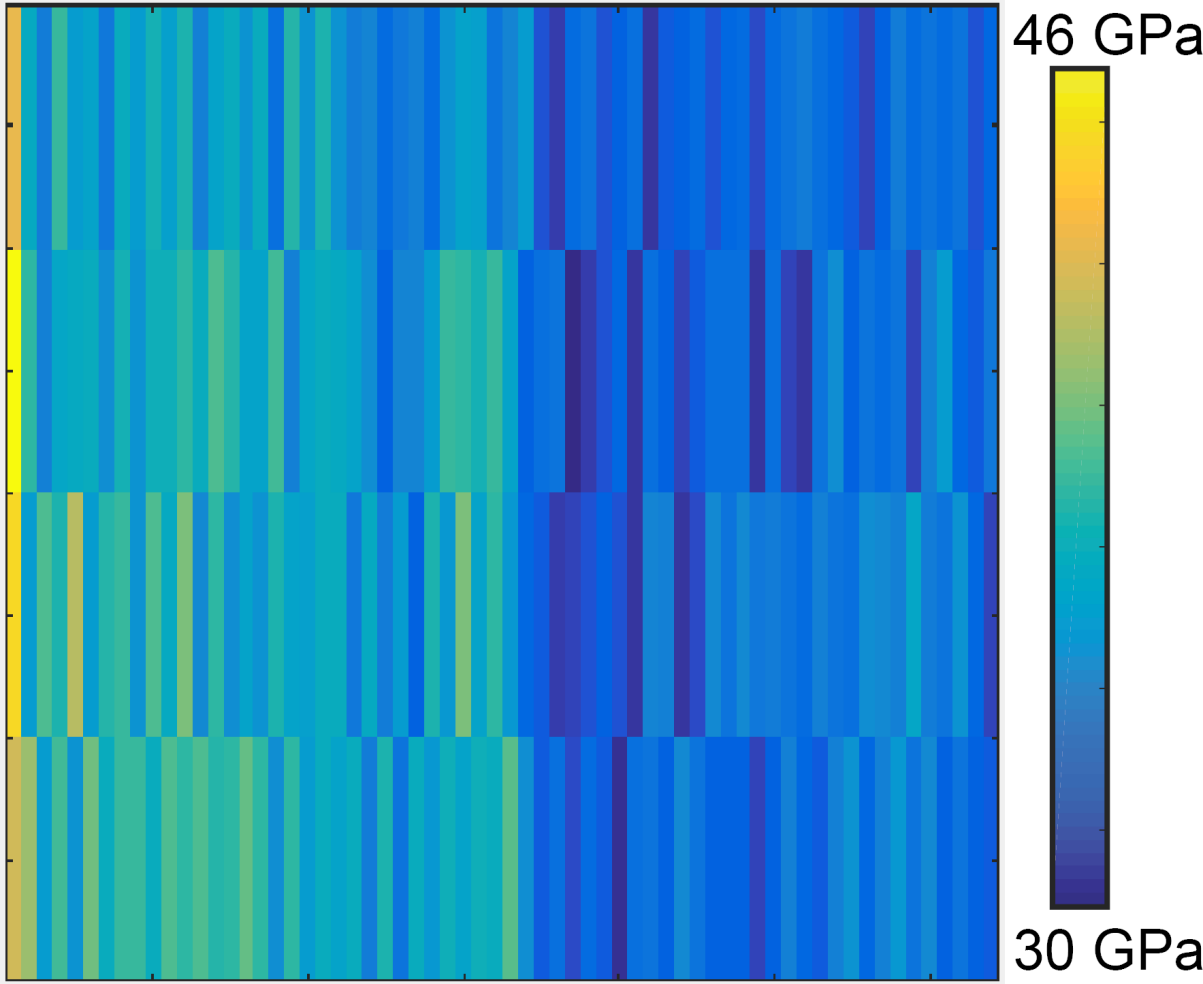
The elastic modulus map according to the grid pattern in four lines, where each line includes 64 rows of static indentation over the HOPG surface.

Figure 12 indicates a map of the elastic modulus over the graphite surface through static indentation. The static indentation has been conducted with a cantilever with stiffness of 38 N/m through a grid pattern over the HOPG surface. This elastic map is a combination of four rows and 64 columns, each comprising a forward and reverse force curve. The resolution of the map is not sufficiently high to see variations in the elastic modulus over a surface step, despite the several hours that it took to acquire this data, in comparison with the minutes it took for the FMM and CR mode images to be acquired. However, the result indicates that the elastic modulus ranges from 30–46 GPa, which is the same elastic modulus range found for HOPG terraces using the soft cantilever and through dynamic AFM measurements presented in this manuscript.
